# EEG Time-Frequency Clustering Reveals Spectral Signatures of Glutamatergic and Cholinergic Activities and Their Interrelations

**DOI:** 10.3390/biomedicines14030669

**Published:** 2026-03-15

**Authors:** Vasily Vorobyov, Alexander Deev

**Affiliations:** 1 Institute of Cell Biophysics, Russian Academy of Sciences, 142290 Pushchino, Russia; 2 Institute of Theoretical and Experimental Biophysics, Russian Academy of Sciences, 142290 Pushchino, Russia

**Keywords:** l-glutamic acid hydrochloride, oxalyldiaminopropionic acid, n-methyl-d-aspartic acid, quisqualic acid, 3-(2 carboxypiperazine-4-yl)-propyl-1-phosphonic acid, glutamic acid diethyl ester, physostigmine sulfate, scopolamine hydrobromide

## Abstract

**Background:** The discovery of electroencephalogram (EEG) biomarkers of direct transmitter–receptor interactions in studies of neurotransmitter mechanisms underlying brain function remains relevant. Recently, EEG “signatures” of monoaminergic systems have been demonstrated using the “time-frequency clustering” approach. In the current study, the glutamic and cholinergic systems were under similar analysis with additional emphasis on their potential interaction. **Methods:** In non-anesthetized freely moving rats, we studied the EEG effects of agonists for glutamate receptors, injected into the cerebral lateral ventricles, and their modification after pretreatment with corresponding antagonists. The same protocol was used for acetylcholine receptors, activating and blocking substances that penetrate the blood–brain barrier (BBB) after subcutaneous injections. A clustering of significant time-dependent changes in tiny frequency subranges of the EEG spectra was performed. **Results:** After injections of agonists for glutamate receptors, two clusters with enhanced and suppressed activities around 2/4 and 10 Hz, respectively, were observed in the EEG spectra. These effects were reduced by pretreatment with corresponding receptor blockers. A cholinomimetic, physostigmine, decreased EEG activity around 2 and 10 Hz and increased near 5 and 22 Hz. Scopolamine, blocking muscarinic cholinoreceptors, weakened the effects of physostigmine. Intracerebral pretreatment with NMDA and AMPA receptor blockers differently modified the effects of physostigmine. The results demonstrate the EEG biomarkers of glutamatergic and cholinergic systems, as well as the specificity of interactions between them at the intracerebral level. **Conclusions:** The developed EEG time-frequency clustering is a potentially useful approach for the clinical evaluation of glutamatergic/cholinergic pathology and its correction by corresponding substances penetrating the BBB.

## 1. Introduction

Enhanced interest in the electroencephalogram (EEG) is associated with its potential in studies of an integration lying between the forms of behavioural organization and neurophysiological phenomena occurring at the level of individual cells and/or their local constellations. This unified approach, applied in both human studies and animal experiments, simplifies the mutual extrapolation of results, particularly those related to the EEG effects of various pharmacologically active substances. Efficacy of the “pharmaco-EEG” [1] is significantly enhanced by using the extensive material on the reflection in EEG of changes in activities of the main cerebral neurotransmitter systems. The methodological basis for these is the determining role of neuronal membrane potentials in the genesis of EEG [2] and their close connection with neurotransmitter processes in the brain [3]. This approach allows filling the gap between empirically accumulated data in the field and those neurophysiological/neurochemical mechanisms underlying changes revealed in the EEG.

The EEG frequency spectrum has been shown to be extremely effective in evaluating the influence of pharmacologically active drugs on brain functioning [4] and is correlated with changes in neurotransmitter receptors, particularly in their density, which are observed in neurodegenerative pathology [5]. Disturbances in activities of the glutamatergic and cholinergic systems have been shown to be associated with dysregulations in emotions and sleep, with the development of depression and stress [6,7,8,9,10]. Overall, the association between the frequency composition of the EEG and glutamatergic/cholinergic system activity has been demonstrated to be a reliable marker in studies of memory and/or mental impairments [11,12] and Alzheimer’s disease (AD) [13]. Given the close interrelation between these neurotransmitter systems [14,15], the EEG spectral analysis is expected to be useful in the evaluation of their contribution to various phenomena, for example, in the induction of bursts [16] closely associated with memory formation [17].

The first step in the realization of this approach is the revealing of neurotransmitter–receptor association of the dose-dependent changes in EEG frequency spectra that might be produced by agonists and antagonists for the receptors studied. Secondly, the EEG effects of the agonists for chosen receptors, which are attenuated or eliminated after treatment with corresponding antagonists, are considered to be directly associated with the transmitter–receptor interaction. Thirdly, hitherto these EEG markers have been studied under complex, indirect modulation of glutamatergic and cholinergic systems, while the revealed variations were explained by those in peculiar pharmacological profiles of applied substances [12,18]. Sometimes this makes it difficult to understand the mechanisms analyzed by the use of drugs with multiple types of pharmacological activity.

It should be mentioned that the correct use of the well-known Fourier transformation for building the EEG frequency spectra needs stationarity of the analyzed signals. However, EEG is well known to be a stochastic process in nature, with enhanced non-stationarity due to internal disturbances and external invasions. In contrast, the periodogram approach [19] is a universal technique for EEG analysis, allowing the revealing of subtle changes in the amplitude–frequency composition of the signal [20], as demonstrated in studies of somnogenic mechanisms [21].

Furthermore, traditional averaging across “classical” frequency ranges (delta, theta, alpha, and beta) frequently affects the EEG spectral mosaic because of its time-dependent fluctuations, resulting in the crossing of the wide frequency band boundaries. A new approach for identifying and clustering specific time-frequency domains of significant changes in the EEG spectra has been developed and successfully applied in our previous study on neurotransmitter–receptor interactions in the monoaminergic systems [22]. Currently, we focus on, firstly, how the brain’s EEG frequency profile is modified by the mediator–receptor interaction in the glutamatergic and cholinergic systems. Secondly, we analyzed whether the glutamate receptor inhibition affects activated cholinergic receptors. The clustering approach based on a refined period–amplitude algorithm with high frequency resolution was used for examination of EEG changes in non-anesthetized freely moving rats after injections of agonists and antagonists for glutamate and acetylcholine receptors. In preliminary studies, dose-dependent EEG characteristics of the analysers were evaluated, followed by special studies with combined administration of an agonist and corresponding antagonist at chosen doses. For the glutamatergic system, intracerebroventricular (i.c.v.) injections of the analysers (agonists and antagonists) for the receptors were used. For the cholinergic system, subcutaneous (s.c.) injections of corresponding substances that bypass the blood–brain barrier (BBB) were chosen, aiming to demonstrate receptor-associated EEG effects of drugs at the periphery route of drug delivery into the brain. Agonist-produced clusters in the EEG spectra, which were reduced by the preliminary injection of the corresponding antagonist, were considered as the specific “markers” of the neurotransmitter–receptor interaction. Thus, a modification of the EEG effects of peripherally applied cholinomimetic (physostigmine) by centrally injected antagonists for glutamatergic receptors allowed the conclusion about the potential interaction between these neurotransmitter systems at the intracerebral level.

## 2. Materials and Methods

### 2.1. Experimental Animals

Sixty-two male Wistar rats from colonies at the University of Glasgow (Scotland, UK) and bred under controlled barrier conditions at the Pushchino Department of the Institute of Bioorganic Chemistry (Pushchino, Russia) were used in this study. The animals were housed with a 12 h/12 h light/dark cycle, 22–25 °C RT, 50–55% relative humidity, with food and water ad libitum. Experiments were conducted between 8:00 a.m. and 5:00 p.m. All manipulations were performed in accordance with the principles enunciated in the “Guide for Care and Use of Laboratory Animals, NIH publication No 85-23”, and with the “Guidelines for accommodation and care of animals and the principles of the Directive 2010/63/EU” on the protection of animals used for scientific purposes. The main effort was made to minimize animal suffering and reduce the number of subjects used.

### 2.2. Electrodes and Cannula Implantation

Adult (8–9-week-old, 290–330 g) rats with electrodes and cannulas implanted in the cortex and lateral cerebral ventricles, respectively, were studied. The implantation procedure was performed under Pentobarbital (Merck KGaA, Darmstadt, Germany) s.c. (60 mg/kg) narcosis with repeated injections (30 mg/kg) if necessary. At sufficient depth of anesthesia (usually 15–20 min after the first injection), the animals were immobilized by a soft medical bandage on the operating table, followed by a treatment of the skin on the head dorsal surface with a local analgesic, ampulated procaine (AdvaCare Pharma, Cheyenne, WY, USA). Two to three minutes later, the upper part of the skull was cleaned of the skin flap and periosteum, treated with hydrogen peroxide and dried. The animals were placed in a modified stereotaxic frame for our aims, and the skull was positioned and fixed according to the requirements of the rat brain atlas [23]. Based on coordinates chosen from this atlas, the holes for the electrodes and intraventricular cannula with diameters of 0.4 mm and 1.2 mm, respectively, were drilled.

Stainless-steel EEG recording electrodes 0.4 mm in diameter were implanted in the right frontal cortex (AP −1.5, L 2 mm) [24]. Similarly prepared reference electrodes were placed in the nasal bone midline, close to the midline at a distance of 8–9 mm in front of the bregma, and at an optimal depth to exclude EEG contamination by respiration/sniffing artefacts. For this, a signal between an electrode temporarily clamped on the ear and the reference electrode was monitored during its implantation. A stainless cannula with 1 mm outer diameter and 11 mm length was placed into the right lateral ventricle with coordinates of AP −0.4, L 3.2, DV 3.7 and an angle of 20° between the cannula and midline vertical plane [23]. At the first step, dental hygroscopic “Zinc-phosphate cement” (“Vladmiva”, Moscow, Russia) was used for fixation of all metallic details to the skull and protection of its surface from the liquor elevated sometimes through the bone sutures. A mini-connector (Sullins Connector Solutions, San Marcos, CA, USA) with soldered electrodes was maintained on the skull using a quick-hardening Noracril 100 (“StomPolimer”, Ufa, Russia).

At the first step of the cannula implantation, the correct positioning was operatively characterized by the release of a liquor drop from its upper tip. Furthermore, angiotensin II (10 μg per rat) injected through the cannula was used to assess the level of water consumption by the animal as an additional indirect test of the cannula positioning [25]. Before the main experiments, with the aim of practicing this implantation procedure, the application of methylene blue solution (6–8 μL per animal) was used [26]. Final morphological control of the cannula positioning in the brain was performed after Nembutal overdose euthanasia on the brain freezing slices.

### 2.3. EEG Recording and Drug Treatment

Each animal was placed in an individual cage to protect it from stressful interactions with other rats and to prevent damage to the implants fixed to the head. Since the fourth day after surgery, every animal was placed in an experimental box (5 × 17 × 20 cm^3^) prepared from transparent Perspex for one hour per day to manipulate the connection/disconnection of the rat to/from the amplifier. One week after surgery, each animal was adapted to the box for 20–30 min, followed by a 10 min baseline EEG recording starting about 20 min after placing the animal in the cage. Afterwards, through the cannula, the injections of a drop (5 µL) of a vehicle (saline) and, in a day, of an agonist or antagonist for the transmitter receptors at different doses were performed (see a list of the drugs used in this study in Table 1). EEG was recorded for 60 min after intracerebroventricular (i.c.v.) injections. It should be mentioned that performing control experiments with saline on the same animal that was used for testing the drugs has an evident advantage. Indeed, this allows avoiding the randomization procedure of rats, i.e., ignoring their individual peculiarities that are well known to be important for similar studies [26].

In a special (“combined”) series of “antagonist + agonist” experiments, an agonist was injected with a 20 min delay, while in control experiments, an antagonist was replaced by saline. In the baseline period, EEG recordings were performed exclusively in relaxed animals with closed eyes, guaranteeing similarity in their initial states in different experimental groups, thus allowing their correct comparison. All i.c.v. injections in a volume of 5 μL were performed slowly (for about 2 min) in non-anesthetized, freely moving animals through the guide cannula by using a Hamilton syringe attached to an elastic tube with a blunt needle (0.8 mm in diameter) at its tip. In advance, the infusion system was treated with ethanol and sterile saline. The freshly prepared agonists and antagonists’ solutions were passed through a Millipore filter (a pore diameter of 0.22 μm) for their sterilization. The needle was left inside the implanted cannula for one to two minutes after injection to allow the residuals of the solution to move into the brain. After that, the needle was carefully replaced by a preliminary sterilized stainless wire, and, finally, the upper tip of the implanted cannula was covered by a sterile plastic lid. As a rapid spread throughout the brain of a compound injected into the lateral ventricle has been shown [27], EEG recordings in rats started immediately after the injection and continued for an hour, predominantly in the awake state. Typically, the full experiment on an individual rat was continued for about two hours.

The same protocol was used in experiments with acetylcholine receptor-activating (physostigmine) and blocking (scopolamine) substances, well penetrating the blood–brain barrier (BBB) at subcutaneous (s.c.) injections. Glutamatergic influence on cholinergic EEG effects was studied in combined experiments where central (i.c.v.) injection of the glutamate receptor antagonists (CPP and GDEE) was followed (with 20 min delay) by periphery (s.c.) applied physostigmine.

### 2.4. Computation of EEG Spectra

The frequency spectra (0.25–30.5 Hz) on-line analysis of 12 s successive EEG fragments was performed by using a modified version of the period–amplitude approach [28]. A sampling rate of 330 Hz was obtained by using a multi-channel A/D DT2814 converter (Data Translation, Inc., Marlboro, MA, USA). This developed programme allows automatic detection of the sampling rate and the maximum number of narrowest frequency bands. In this study, sums of EEG amplitudes in twenty tiny frequency subranges and amplitude ratios for each of them over the sum of all amplitudes in the chosen range (0.25–30.5 Hz) were estimated. Furthermore, the program automatically rejected incorrect EEG fragments to meet a high-quality criterion for the EEG recordings, the primary criterion for considering the data obtained from an individual animal. It might be noted that this option of the program was used very rarely due to the development of special technical facilities: tight plugging of the cable connecting a rat brain to a digital Neuro-MEP amplifier (Neurosoft Ltd., Ivanovo, Russia) and covering the cable by a flexible shield (see [22] for details).

### 2.5. Statistics

Summed amplitudes in the narrow subranges of the EEG spectra obtained for all rats from each group in sessions with an analyzed drug and a vehicle (saline) were calculated as [(drug–vehicle)/vehicle] × 100%. Significance (*p* < 0.05) of visible differences was assessed by the non-parametric Mann–Whitney U-test. The image processing algorithms were applied to evaluate the revealed effects in the time-frequency matrices built by the developed “clustering” program. To achieve this aim, the resizing of the distributions of calculated values was performed using bilinear interpolation, and the smoothening was achieved through two-dimensional Fourier filtration. The areas (“clusters”) for both positive and negative significant values were then selected by building equipotential edge lines in each narrow frequency sub-band. For each of the “clusters”, their average parameters were estimated, including mean value, area, size and the time-frequency position calculated as a centre of mass of an interior value distribution. To compare the significance (*p* < 0.05) of differences in the “clusters” effects of different drugs, the non-parametric Mann–Whitney U-test was used. The dose-dependence approach applied in this study was able to reveal different (total and peculiar) influences of the drugs studied on the time-frequency “clustering” in the EEG spectra, highlighting its potential for further analysis in this area. For U-test analyses and calculation of power and effect size, STATISTICA 10 (StatSoft, Inc., Tulsa, OK, USA) and G*Power 3.1.9.4 (assessed on 6 February 2019), respectively, were used. G*Power indicated that an effect size of 0.75–0.9 and a power of 0.8 were optimal for the chosen sample sizes in our EEG study on different animal groups (see below).

## 3. Results

### 3.1. Glutamatergic Reception

**Figure 1:**
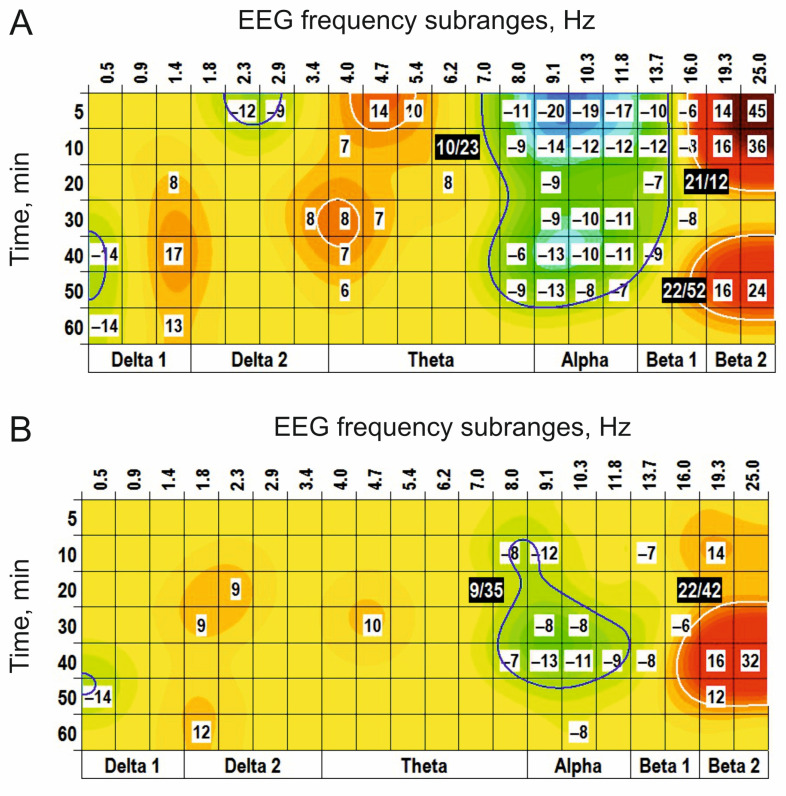
EEG effects of intraventricular injections of L– and D–glutamate ((**A**,**B**), respectively, 10 nmol, *n* = 5, for both) vs. saline. The main parameters of the (−) and (+) clusters outlined by blue and white color, respectively, are denoted numerically as a “centred frequency/time” in black coloured rectangles.

Centrally (i.c.v.) applied L–glutamate significantly suppressed EEG oscillations in the frequency subranges centred at 10 Hz (“(−)” cluster) and two (+) clusters centred at 21 Hz and 22 Hz (Figure 1A). At the same dose, D–glutamate (inactive isoform of glutamate) initiated evidently less powerfully expressed clusters (Figure 1B).

**Figure 2:**
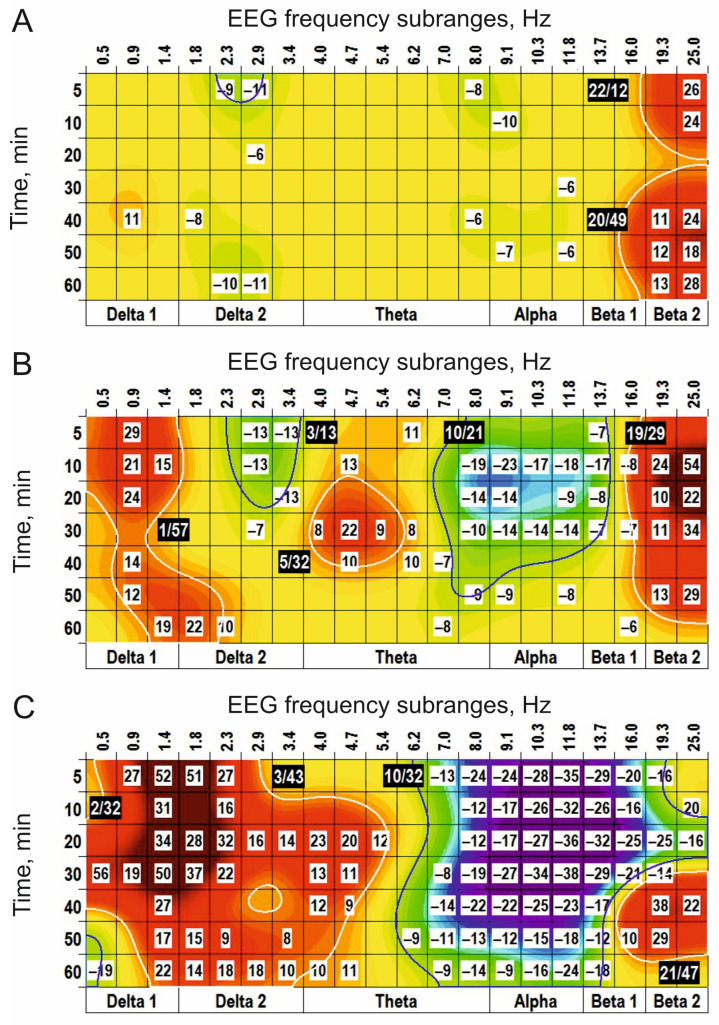
EEG effects of intraventricular injections of ODAP at different doses ((**A**–**C**): 1, 10, and 100 nmol, *n* = 5, 7, and 6, respectively) vs. saline. The main parameters of the (−) and (+) clusters outlined by blue and white color, respectively, are denoted numerically as a “centred frequency/time” in black coloured rectangles.

Another structural analogue of glutamate, ODAP, produced two (+) clusters centred at 20/22 Hz (Figure 2A). At a higher dose of 10 nmol, ODAP expanded the effects on the other time-frequency ranges in EEG spectra: additional (+) clusters around 1 Hz and 5 Hz and (−) clusters centred at 3 Hz and 10 Hz were observed (Figure 2B). The highest dose of ODAP (100 nmol) was characterized by widening and prolongation of the effects (Figure 2C).

**Figure 3:**
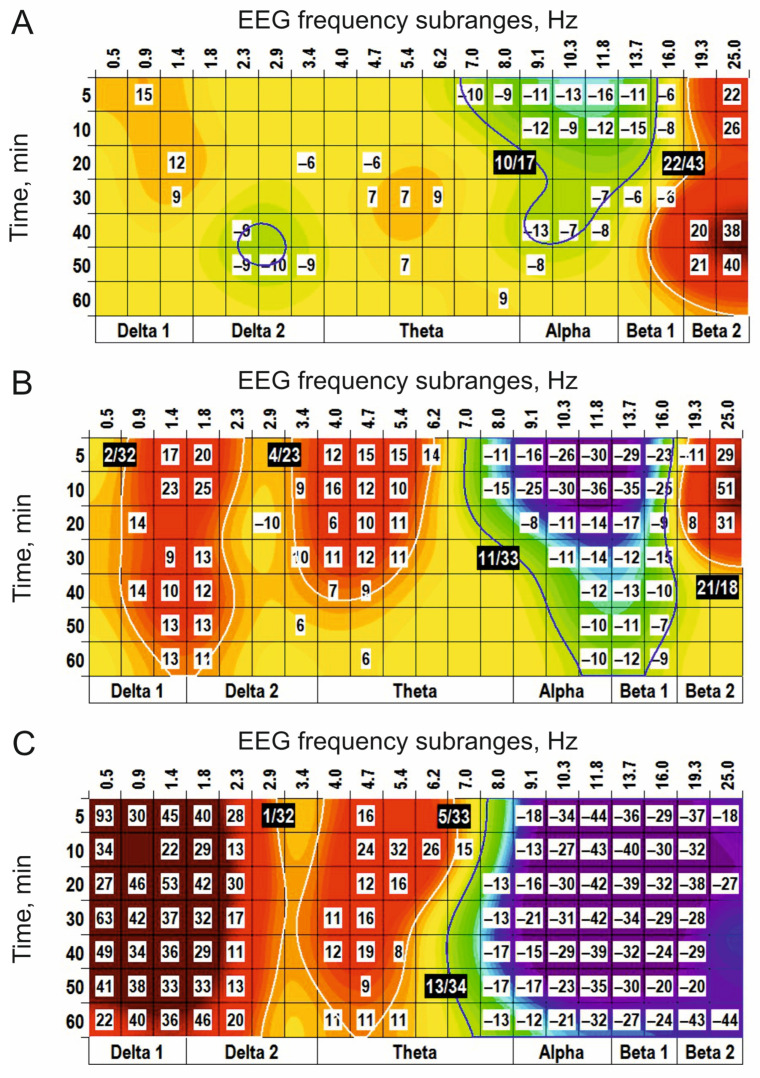
Clasterization of the EEG spectra after central applications of NMDA at different doses (**A**–**C**): 0.3, 1, and 10 nmol, respectively, *n* = 5, for all) vs. saline. The main parameters of the (−) and (+) clusters outlined by blue and white color, respectively, are denoted numerically as a “centred frequency/time” in black coloured rectangles.

Centrally injected NMDA, one of the potential agonists for glutamate receptors, initiated the clusters in the EEG spectra (Figure 3A) similar to those observed after L-glutamate (Figure 1A).

**Figure 4:**
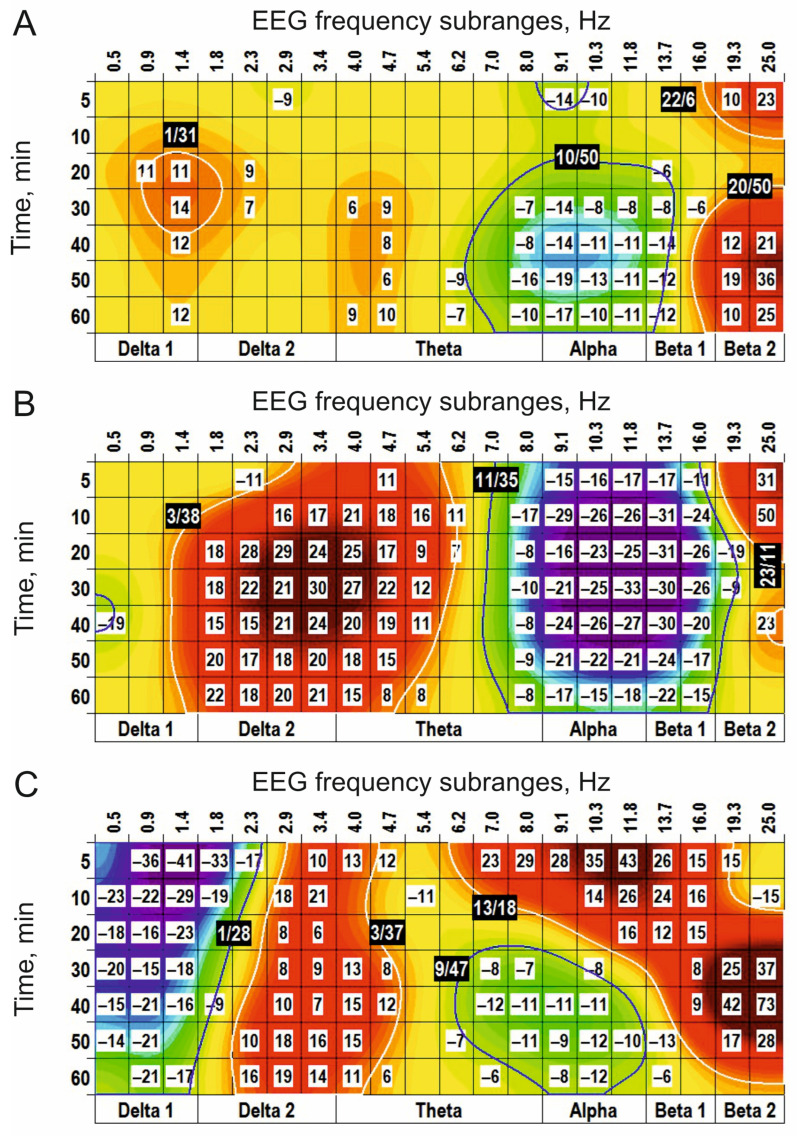
Clasterization of the EEG spectra after central applications of CPP at different doses ((**A**,**B**): 0.1 and 5 nmol, respectively; *n* = 5, for both) vs. saline. In (**C**), an inversion of the clusters with centres at 2 Hz and 11 Hz characteristic of NMDA alone (see Figure 3B) after pretreatment with CPP (0.3 nmol, *n* = 5) is shown. These highlight the involvement of NMDA transmission in modulating the EEG signal that forms these clusters. The main parameters of the (−) and (+) clusters outlined by blue and white color, respectively, are denoted numerically as a “centred frequency/time” in black coloured rectangles.

At higher doses, NMDA produced additional (+) clusters around 2 Hz and 4 Hz and significantly enlarged (−) clusters around 11 Hz (Figure 3B). At the highest dose (10 nmol), the effects of the agonist were widened and prolonged (Figure 3C). A specific antagonist for NMDA receptors, CPP, produced (−) cluster and (+) cluster in the EEG spectra centred at 10 Hz and 20 Hz, respectively, and with a relatively long (50 min) delay (Figure 4A). At the highest dose, CPP significantly increased the (−) cluster. In addition, a new powerful (+) cluster centred at 3 Hz was denoted (Figure 4B). CPP applied before NMDA (Figure 4C) inverted both the (+) cluster and the (−) cluster centred at 1 Hz and 13 Hz, which were characteristic of NMDA used alone (Figure 3C). The results obtained highlight a role of NMDA synaptic mechanisms in the generation/modulation of these EEG oscillations.

**Figure 5:**
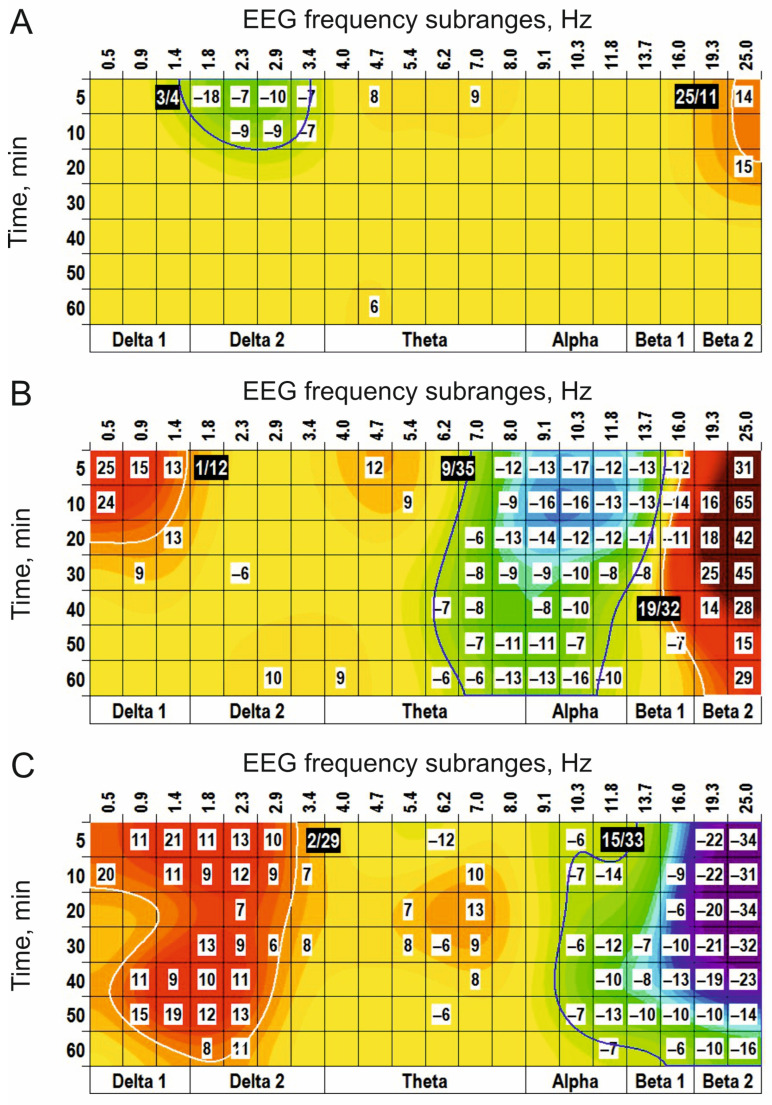
Clasterization of the EEG spectra after central applications of quisqualate at different doses ((**A**–**C**): 0.1, 1, and 5 nmol; *n* = 5, 5, and 7, respectively). The main parameters of the (−) and (+) clusters outlined by blue and white color, respectively, are denoted numerically as a “centred frequency/time” in black coloured rectangles.

Centrally injected another glutamate receptor agonist, quisqualate, was practically ineffective at a low dose (0.1 nmol) (Figure 5A), while at the higher dose (1 nmol), it produced centred at 1 Hz very small (+) cluster, highly expressed (−) cluster around 9 Hz and (+) cluster at 19 Hz, respectively (Figure 5B). At the highest dose (5 nmol), quisqualate significantly widened and extended the latter cluster and shifted the (−) cluster to the higher frequencies, replacing the (+) cluster (c.f., Figure 5B,C).

**Figure 6:**
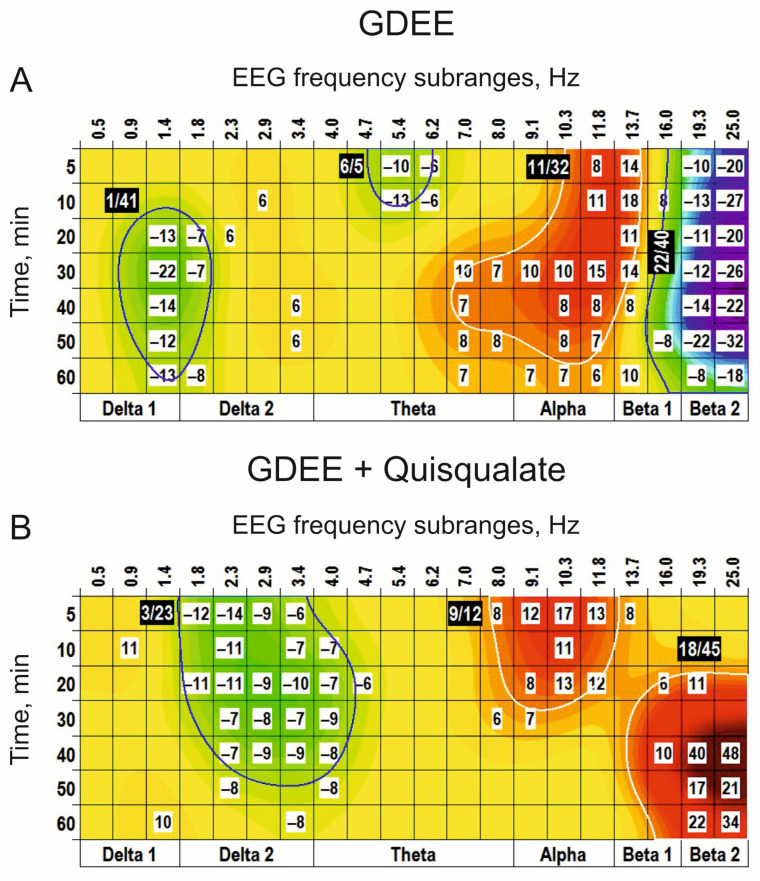
Clasterization of the EEG spectra after central applications of GDEE ((**A**): 10 nmol; *n* = 7) vs. saline. In (**B**), the modified clusters centred at 1 Hz and 9 Hz, characteristic of quisqualate alone (see Figure 5B), after pretreatment with GDEE (5 nmol, *n* = 5), are shown. These highlight an involvement of AMPA receptors in the modulation of the EEG signal forming these clusters. The main parameters of the (−) and (+) clusters outlined by blue and white color, respectively, are denoted numerically as a “centred frequency/time” in black coloured rectangles.

Centrally injected GDEE (10 nmol), an antagonist for quisqualate receptors, produced three main clusters in the EEG spectra: (−) cluster centred at 1 Hz, (+) cluster around 11 Hz, and (−) cluster centred at 22 Hz (Figure 6A). Pretreatment with GDEE (5 nmol) significantly modified the EEG effects of quisqualate applied alone (see Figure 5B), forming a (−) cluster centred at 3 Hz and replacing the (−) cluster centred at 9 Hz by the (+) cluster centred at the same frequency (c.f., Figure 5B and Figure 6B). Interestingly, GDEE pretreatment potentiated the quisqualate effect in the beta range.

### 3.2. Cholinergic Reception

**Figure 7:**
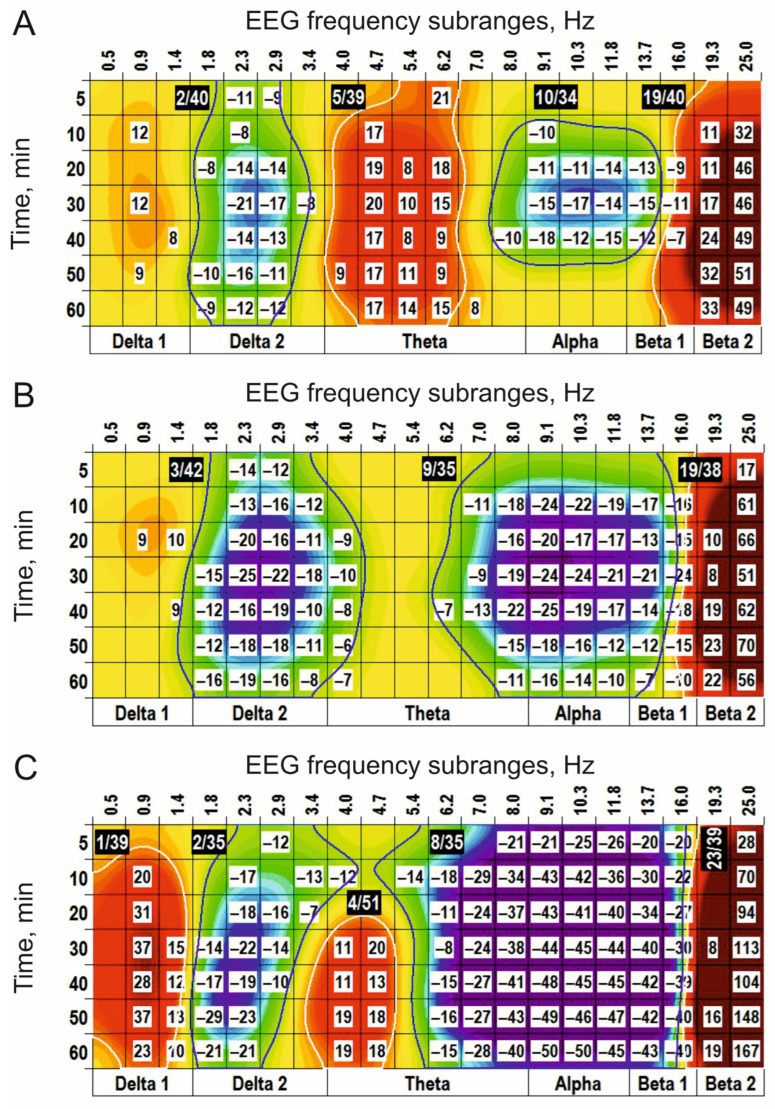
Clasterization of the EEG spectra after subcutaneous application of physostigmine at different doses ((**A**–**C**): 0.05, 0.25, and 1 mg/kg, respectively; *n* = 7, for all) vs. saline. The main parameters of the (−) and (+) clusters outlined by blue and white color, respectively, are denoted numerically as a “centred frequency/time” in black coloured rectangles.

**Figure 8:**
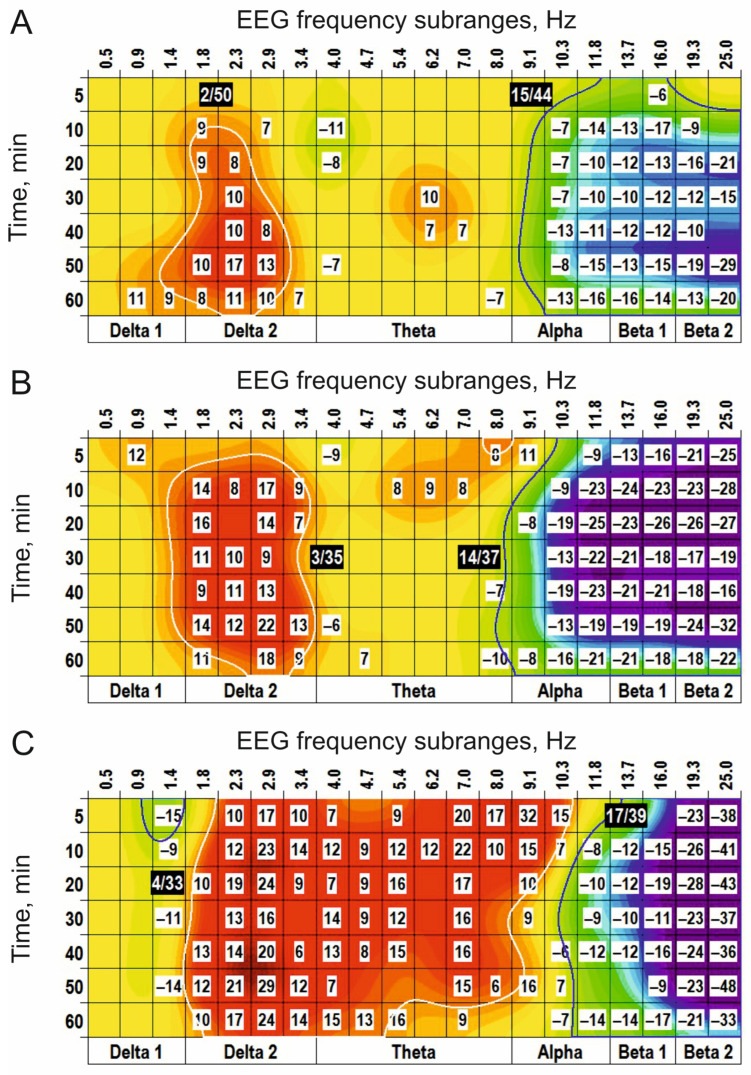
Clasterization of the EEG spectra after subcutaneous application of scopolamine at different doses ((**A**–**C**): 0.5, 2, and 10 mg/kg, respectively; *n* = 4, for all) vs. saline. The main parameters of the (−) and (+) clusters outlined by blue and white color, respectively, are denoted numerically as a “centred frequency/time” in black coloured rectangles.

**Figure 9:**
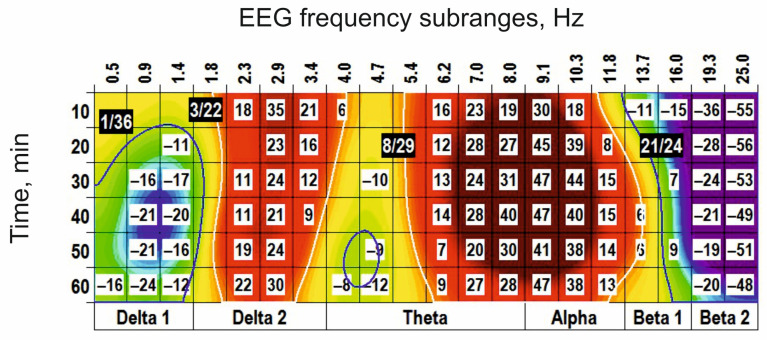
An inversion of the physostigmine-produced clusters centred at 3 Hz, 9 Hz, and 19 Hz (see Figure 7B) after periphery injection of scopolamine (s.c., 0.5 mg/kg, *n* = 6) is shown. These highlight an involvement of cholinergic transmission in the modulation of EEG oscillations forming these clusters. The main parameters of the (−) and (+) clusters outlined by blue and white color, respectively, are denoted numerically as a “centred frequency/time” in black coloured rectangles.

Physostigmine, prolonging the transmitter action of acetylcholine in the synaptic cleft, s.c. injected at a dose of 0.05 mg/kg, initiated in the EEG spectra two (−) clusters centred at 2 Hz and 10 Hz and two (+) clusters centred around 5 Hz and 19 Hz (Figure 7A). At the higher dose (0.25 mg/kg), physostigmine enlarged both (−) clusters and only the (+) cluster around 19 Hz, while eliminating the (+) cluster centred at 5 Hz (c.f., Figure 7A,B). At the highest dose (1 mg/kg), physostigmine predominantly potentiated the (−) cluster centred at 8 Hz and the (+) cluster centred at 23 Hz (Figure 7C). An antagonist for cholinergic receptors, scopolamine (0.5 mg/kg), produced in the EEG spectra the (−) cluster centred at 2 Hz and the (+) cluster centred at 15 Hz (Figure 8A). At the higher doses (2 mg/kg and 10 mg/kg (Figure 8B and Figure 8C, respectively)), scopolamine significantly increased the cluster sizes. Pretreatment with scopolamine (Figure 9) produced an inversion of the main effects of physostigmine applied alone (Figure 7B). The results obtained highlight a role of cholinergic synaptic mechanisms in the generation/modulation of the EEG oscillations in frequency subranges centred at 3 Hz, 8–9 Hz, and 19 Hz.

### 3.3. Glutamatergic and Cholinergic Receptors Interrelation

**Figure 10:**
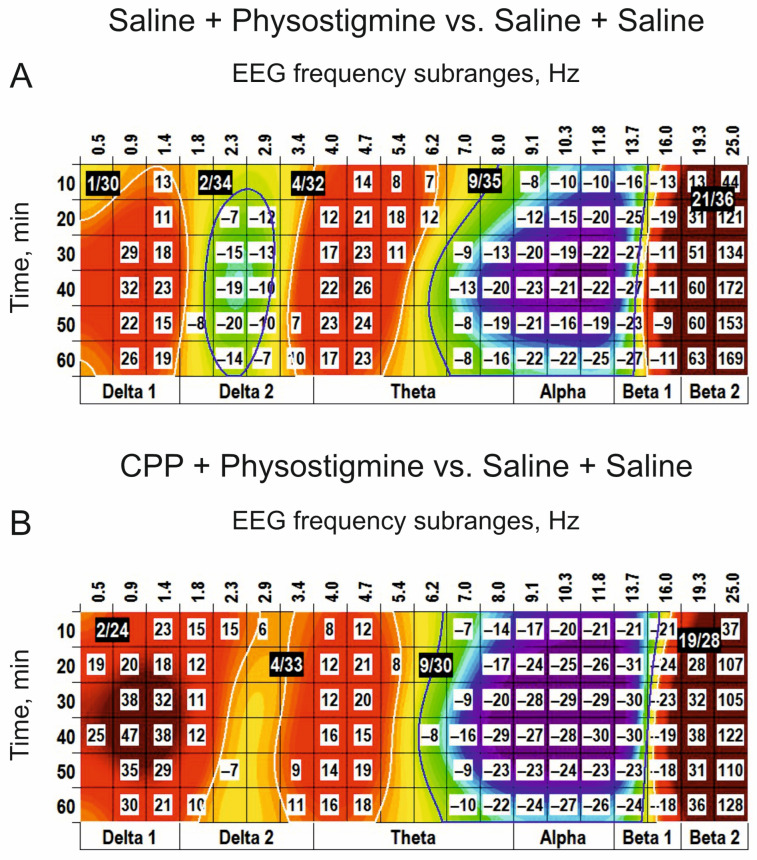
EEG clusterization effects of periphery application of physostigmine (s.c., 0.2 mg/kg) and their evident modification by central (i.c.v., 0.1 nmol) pretreatment with CPP ((**A**,**B**), respectively; *n* = 7, for both) vs. saline. The main parameters of the (−) and (+) clusters outlined by blue and white color, respectively, are denoted numerically as a “centred frequency/time” in black coloured rectangles.

In “combined” experiments with preliminary i.c.v. injection of CPP (0.1 nmol), blocking NMDA receptors, and s.c. injection of a cholinomimetic, physostigmine (0.2 mg/kg), functional interrelations between receptors for glutamate and acetylcholine were analyzed (Figure 10). The main effects of physostigmine after i.c.v. injection of saline (Figure 10A) were similar to those observed after periphery injection of physostigmine alone (c.f., Figure 7) and characterized by the (+) clusters centred at 1 Hz, 4 Hz, and 21 Hz and the (−) clusters centred at 2 Hz and 9 Hz. CPP produced widening of the (+) cluster centred at 1 Hz at the expense of the neighbouring (−) cluster, tended to amplify the (−) cluster centred at 9 Hz and attenuated the (+) cluster centred at 19 Hz (Figure 10B).

**Figure 11:**
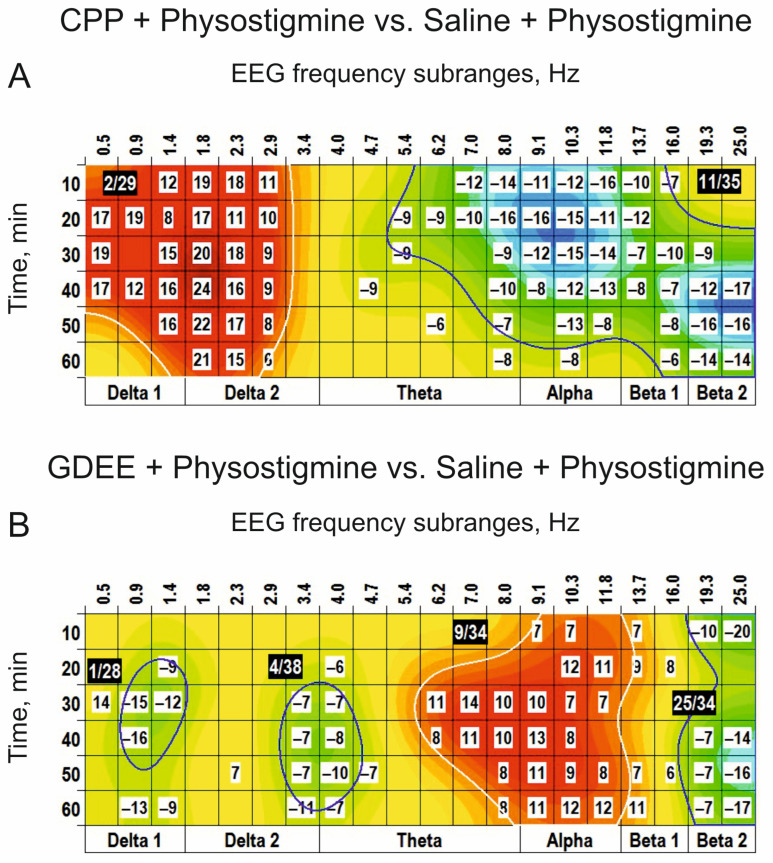
Modification of the EEG effects of periphery application of physostigmine (s.c., 0.2 mg/kg) by central pretreatment with CPP and GDEE ((**A**,**B**), i.c.v., 0.1 and 10 nmol, respectively; *n* = 7, for both). CPP attenuates the physostigmine-produced clusters centred at 2 Hz and 19 Hz. GDEE attenuates the physostigmine-produced clusters centred at 3 Hz, 10 Hz and 19 Hz. These highlight a specific role of different subtypes of glutamate receptors, NMDA and AMPA, in the EEG effects of a cholinomimetic, physostigmine. The main parameters of the (−) and (+) clusters outlined by blue and white color, respectively, are denoted numerically as a “centred frequency/time” in black coloured rectangles.

This CPP influence is evidently demonstrated at the direct comparison of the physostigmine effects after i.c.v. injections of CPP and saline (Figure 11A). GDEE pretreatment (10 nmol, i.c.v.) inverted the (−) cluster centred at 9 Hz observed after injection of physostigmine, with additional inversion of the (+) cluster centred at 19 Hz (c.f., Figure 7B and Figure 11B). In contrast to CPP, GDEE significantly attenuated the (−) cluster centred at 3 Hz. These highlight a specific role of different subtypes of glutamate receptors, NMDA and AMPA, in the EEG effects of a cholinomimetic, physostigmine, and, as a whole, a specificity of interaction between glutamatergic and cholinergic mechanisms at the intracerebral level.

## 4. Discussion

In non-anesthetized freely moving rats, a recently developed “time-frequency” clustering technique was used to study the EEG effects of pharmacological activation by agonists and inhibition by antagonists of glutamate and acetylcholine receptors. Receptor agonists formed various clusters of significant deviations in the EEG spectra, with several specific within them. Those clusters, which were diminished/inverted by pretreatment with corresponding antagonists, were considered as the “signatures” of the neurotransmitter system. Furthermore, pretreatment with different antagonists for glutamate receptors has been revealed to affect specifically the clusters associated with cholinergic system activation.

### 4.1. Glutamatergic Transmission

In the EEG spectra, all studied agonists for glutamate receptors produced the (−) clusters in the alpha band with potential flanks in the theta and beta_1_ bands depending on the doses used (c.f., Figure 1A, Figure 2B, Figure 3A,B and Figure 5B, for L-glutamate and ODAP; NMDA and quisqualate, respectively). In parallel with these effects of the agonists, the (+) clusters centred at about 2 Hz were observed. Interestingly, despite the antagonist for NMDA receptors, CPP, producing similar effects (c.f., Figure 3B and Figure 4B), its preliminary injection inverted both the EEG effects of NMDA applied 20 min later (Figure 4C). Thus, a combination of the clusters centred at the mentioned frequencies of the EEG spectra is evidently associated with specific activation of NMDA receptors. In contrast, the (+) cluster centred around 4 Hz seems to be a non-specific additive response (Figure 4C) on both activation and inhibition of these receptors by NMDA and CPP, respectively. Pretreatment with the antagonist for quisqualate receptors, GDEE, inverted the clusters in alpha and beta_2_ bands produced by quisqualate applied alone (c.f. Figure 5B and Figure 6A). Moreover, these frequency areas were affected by GDEE pretreatment (Figure 6B), which together evidently demonstrates a specific involvement of the quisqualate subtype of glutamate receptors in the modulation of high-frequency EEG oscillations. Thus, the use of this EEG “clustering” approach is evidently consistent with that which has recently been developed for an analysis of common pathological pathways at neuropsychiatric illnesses, in particular, schizophrenia [29,30]. Revealed in our study, the association between the EEG clusters and activities of different glutamate receptors, which have been shown to be closely associated with this disease [31], allows the monitoring of their neurotransmitter functions during therapy. The efficacy of our EEG “clustering” approach may be additionally enhanced by its expectable association with excitation/inhibition balance in the EEG, reflecting in turn a ratio of glutamate and GABA levels [32].

### 4.2. Cholinergic Transmission

The cholinomimetic, physostigmine, applied at the mid dose of 0.25 mg/kg, was characterized in the EEG spectra by two (−) clusters centred at 3 Hz and 8–9 Hz and the (+) cluster around 19 Hz (Figure 7B). Pretreatment with scopolamine, the blocker of muscarinic cholinoreceptors, inverted this physostigmine effect at a minimally used dose of 0.5 mg/kg (Figure 9), which highlights the role of the cholinergic receptors in the generation/modulation of these EEG oscillations. Thus, our “clustering” approach might be an additional tool for the recently developed EEG-based “ACh index” considered as a biomarker of cholinergic system functioning, in particular in Alzheimer’s disease and “Lewy bodies” dementia [33]. Furthermore, the “clustering” protocol, including the detailed analysis of dose-dependence separately for agonists and antagonists and their interaction, has potential for use in studies of novel cholinergic receptor modulators and the correlation between EEG and pharmacokinetics [34]. The unveiled “clustering” effects of physostigmine and scopolamine in the beta_2_ band (Figure 3 and Figure 4) are consistent with multiple pieces of evidence indicating a close association between cholinergic system activation and EEG “desynchronization” (see, e.g., [35]). However, the sources of the clusters observed in other time-frequency domains need to be analyzed, given possible involvement of the ionotropic glutamate receptors, NMDA and AMPA, in cholinergic system activity modulation [36].

### 4.3. Glutamatergic–Cholinergic Interaction

Indeed, pretreatment with CPP in our study inverted the EEG effects of physostigmine observed in the delta_2_ and beta_2_ frequency bands (c.f., Figure 7B and Figure 10A) without significant influence on the (+) cluster around 9 Hz. In contrast, preliminarily injected GDEE attenuated/inverted physostigmine produced the (−) and (+) clusters centred at 9–10 Hz and 19 Hz, respectively. Taken together, the effects unveiled in our study by the EEG clustering demonstrate the interaction between glutamatergic and cholinergic systems at the intracerebral level and a specific involvement of different ionotropic glutamate receptors in this interaction, which are widespread throughout the brain [14]. The role of the synaptic activity of muscarinic cholinoreceptors in the emergence of cortical EEG oscillations has been demonstrated, in particular, in isolated neuronal slices [37]. This allowed the authors to make a conclusion about the initiation of the brain waves in wide frequency ranges by random synaptic events in single cholinergic neurons. Revealed in our study is an interaction between receptors for glutamate and acetylcholine that demonstrates an inhibitory influence of spontaneous/baseline glutamatergic system activity on cholinergic transmission. On the other hand, these systems have been shown to affect the bursting of the midbrain dopamine neurons, representing an important rewarding signal [16]. Thus, different brain functions and EEG spectra may be formed by unique compositions of the activities of different neurotransmitter systems. In particular, an acute blockade of NR2C/D subunit-containing NMDA receptors has been shown to decrease sleep spindle oscillations in mice [7], which was observed in our experiments in rats with NMDA receptors blocked by CPP (see Figure 4A,B). However, a similar effect was produced by the glutamate receptor agonists alone, which was characterized by significantly amplified delta and theta waves (see Figure 1A, Figure 2B,C, Figure 3A,B and Figure 5B). Thus, taking into account the evident domination of the “fast” (beta) EEG, these allow the suggestion that the functional state of these animals during the experiments was predominantly formed by activation mechanisms with temporal involvement of relaxation ones.

Given that the glutamatergic–cholinergic interaction may be involved in the regulation of a row of diseases, the developed EEG clustering and other approaches associated with EEG parametrization open an avenue for multifaceted pharmacological corrections of pathological processes that deserve to be reviewed in detail in further studies. Finally, the specificity of AMPA and NMDA receptors revealed in our and other (e.g., [38]) studies may be useful in choosing adequate treatments for different pathologies [39].

## 5. Conclusions

Central injection of glutamate receptor agonists (L-glutamate, ODAP, NMDA, and AMPA) into the brain, lateral ventricles, and periphery (subcutaneous) application of a cholinomimetic, physostigmine, produced time-dependent, significant changes in the cortical EEG frequency spectra in rats. These changes formed areas (“clusters”) whose characteristics were specifically related to the types of studied receptors. Those agonist-produced clusters which were diminished by the preliminary injection of corresponding antagonists (CPP and GDEE, for glutamate receptors, and scopolamine, for cholinoreceptors) were considered as specific EEG “signatures” of the receptors. The results obtained demonstrate a clinical potential of the “EEG clustering” approach for analyzing dysregulation in neurotransmitter mechanisms in treating diseased brains with substances that penetrate the BBB. Thus, the clustering features characterizing the specific interactions between the neurotransmitter systems widen the analytical potential of the developed approach for more detailed analyses of the mechanisms underlying brain pathologies and their treatment.

## Figures and Tables

**Table 1:** The list of substances.

Substances-Analyzers for Different Receptors		
Full Name	Short Name	Affinity
Glutamatergic Receptors		
L-glutamic acid hydrochloride	L-Glutamate	Agonist (Glu)
Oxalyldiaminopropionic acid	ODAP	Agonist (Glu)
N-Methyl-D-aspartic acid	NMDA	Agonist (NMDA)
(+)-Quisqualic acid	Quisqulate	Agonist (AMPA)
3-(2 carboxypiperazine-4-yl)-propyl-1-phosphonic acid	CPP	Antagonist (NMDA)
Glutamic acid diethyl ester	GDEE	Antagonist (AMPA)
Cholinergic Receptors		
(−)-Physostigmine sulfate	Physostigmine	Agonist (ACh)
(−)-Scopolamine hydrobromide	Scopolamine	Antagonist (ACh)

Sources: Glutamate is from Reanal (Telepes, Budapest, Hungary); ODAP is from ACS (Washington, DC, USA); NMDA, quisqualate, CPP, and GDEE are from Sigma-Aldrich (St. Louis, MO, USA); physostigmine is from BOC Sciences (Shirley, NY, USA); scopolamine is from Boehringer Ingelheim Pharma (Ingelheim/Rhein, Germany).

## Data Availability

All data are presented in this paper and numerically will be made available on request.
